# COVID-19 Vaccine Effectiveness during a Prison Outbreak when Omicron was the Dominant Circulating Variant—Zambia, December 2021

**DOI:** 10.4269/ajtmh.22-0368

**Published:** 2022-09-12

**Authors:** John Simwanza, Jonas Z. Hines, Danny Sinyange, Nyambe Sinyange, Chilufya Mulenga, Sarah Hanyinza, Patrick Sakubita, Nelia Langa, Haggai Nowa, Priscilla Gardner, Ngonda Saasa, Graham Chitempa, James Simpungwe, Warren Malambo, Busiku Hamainza, Peter J. Chipimo, Nathan Kapata, Muzala Kapina, Kunda Musonda, Mazyanga Liwewe, Consity Mwale, Sombo Fwoloshi, Lloyd B. Mulenga, Simon Agolory, Victor Mukonka, Roma Chilengi

**Affiliations:** ^1^ Zambia Field Epidemiology Training Programme, Lusaka, Zambia;; ^2^ National Malaria Elimination Centre, Lusaka, Zambia;; ^3^ Centers for Disease Control and Prevention, Lusaka, Zambia;; ^4^ Zambia National Public Health Institute, Lusaka, Zambia;; ^5^ Zambia Correctional Services, Lusaka, Zambia;; ^6^ Lusaka District Health Office, Lusaka, Zambia;; ^7^ University of Zambia, School of Veterinary Medicine, Department of Disease Control, Lusaka, Zambia;; ^8^ Lusaka Province Health Office, Lusaka, Zambia;; ^9^ Ministry of Health, Lusaka, Zambia;; ^10^ Republic of Zambia State House, Lusaka, Zambia

## Abstract

During a COVID-19 outbreak in a prison in Zambia from December 14 to 19, 2021, a case–control study was done to measure vaccine effectiveness (VE) against infection and symptomatic infection, when the Omicron variant was the dominant circulating variant. Among 382 participants, 74.1% were fully vaccinated, and the median time since full vaccination was 54 days. There were no hospitalizations or deaths. COVID-19 VE against any SARS-CoV-2 infection was 64.8%, and VE against symptomatic SARS-CoV-2 infection was 72.9%. COVID-19 vaccination helped protect incarcerated persons against SARS-CoV-2 infection during an outbreak while Omicron was the dominant variant in Zambia. These findings provide important local evidence that might be used to increase COVID-19 vaccination in Zambia and other countries in Africa.

## INTRODUCTION

The highly transmissible Omicron SARS-CoV-2 variant emerged in late 2021, rapidly spreading to many countries and leading to record-breaking case counts globally. In early December 2021, Zambia recorded a rapid rise in confirmed COVID-19 cases, coinciding with confirmation of the Omicron variant in the country. Zambia recorded a daily average of 1,422 confirmed COVID-19 cases in December 2021, compared with an average 15 cases daily the previous month, in November 2021. From December 4 to 24, 2021, all sequenced specimens in Zambia were of the Omicron variant.^1^

Congregate living settings, including prisons, are at high risk for COVID-19 outbreaks.^2^ Congested living conditions make physical distancing and avoidance of crowds challenging in many prison settings. On December 14, 2021, after not recording any COVID-19 cases for months, a local prison in Lusaka noted an increase in the number of residents with respiratory symptoms, 19 of whom tested positive for COVID-19 with rapid diagnostic tests (RDTs). The prison notified the Lusaka District Health Office on the same day and an outbreak investigation was initiated. Mitigation measures were reinforced, including mask distribution and mandatory masking, cohorting persons testing positive, provision of hand hygiene stations throughout the prison, and offering vaccination to unvaccinated/partially vaccinated persons. Of 767 persons incarcerated at the facility during the outbreak, 241 (31.4%) tested positive for COVID-19 from December 14 to 19, 2021.

COVID-19 vaccines have shown remarkable effectiveness, particularly for reducing COVID-19 severity.^3^ COVID-19 vaccine effectiveness (VE) against the Omicron variant is lower than against other variants,^4^^,^^5^ although data from the African continent are all from one country (South Africa).^6^^–^^8^ We measured VE among incarcerated persons during this outbreak in Zambia.

## METHODS

A case–control study of SARS-CoV-2 infection and symptomatic infection by vaccination status was conducted among persons incarcerated at the prison facility during the outbreak from December 14 to 19, 2021. The facility is in Lusaka, the national capital of Zambia, and houses men aged ≥ 13 years awaiting court cases or transfer to other prisons after sentencing. The prison has five cells built to house 92 male persons, although at the outbreak onset 767 persons were incarcerated (i.e., > 800% beyond capacity). In Zambia, incarcerated persons are tested for COVID-19 upon admission to the prison.

During the outbreak, all incarcerated persons were retested for COVID-19 using Panbio COVID-19 Ag rapid test (Abbott Rapid Diagnostics, Jena, Germany) from December 14 to 19, 2021. Cases and controls were incarcerated persons testing COVID-19 positive and negative, respectively. Participants were recruited into the study from December 20 to 24, 2021, by reviewing the outbreak line list maintained by the prison health team and district health office, attempting to frequency match controls by cases’ cell assignment. Verbal consent was obtained from the participants, and for minors aged 13 to 17 years consent was obtained from the prison warden as per Zambia Correctional Services policy. This study was approved by the ERES Ethics review board and National Health Research Authority. The activity was reviewed by the US Centers for Disease Control and Prevention and was conducted consistent with applicable federal law and CDC policy.

A standardized questionnaire that included information on demographics and medical history, including COVID-19 vaccination and test results was administered by trained interviewers. Self-reported COVID-19 test results were confirmed with the line list where the RDT results were recorded. Vaccination status was cross-referenced with the national registry.

Full vaccination was defined as having completed a full primary series of COVID-19 vaccine ≥ 14 days before COVID-19 testing (i.e., a single dose of Janssen vaccine or two doses of AstraZeneca, Pfizer-BioNTech, Moderna, or Sinopharm). Partial vaccination was defined as having received the first dose of a two-dose vaccine ≥ 14 days before COVID-19 testing but not the second dose or receiving the second dose ≤ 13 days before testing. Multivariable logistic regression was used to calculate the odds of SARS-CoV-2 infection and symptomatic infection, adjusting for age, number of comorbidities, jail cell, and self-reported mask use. VE was calculated as 1 minus the adjusted odds ratio times 100. To reduce the risk of misclassification bias related to false-negative RDT test results, we conducted a sensitivity analysis excluding symptomatic controls.

## RESULTS

In total, 385 (50.2%) of the 767 incarcerated persons present during the outbreak were reached for interview, and 382 (49.8%) consented to being enrolled in the study. All were males, with a median age of 28 years (interquartile range: 21**–**36 years) (Table 1). Overall, 84 (22.0%) had at least one comorbidity, with HIV being most common (*N* = 40, 10.5%). Only 16 (4.1%) reported having had COVID-19 previously.

**Table 1:** Characteristics and outcomes of cases and controls during an COVID-19 outbreak in a prison in Zambia, December 2021

Variable	Cases* (*N* = 180)	Controls* (*N* = 202)	*P* value†
Age, median years (IQR)	27 (21–36)	28 (21–36)	0.58
Imprisonment duration, median days (IQR)	41 (13–96)	43 (17–120)	0.79
Education level, *n* (%)			
None	5 (2.8)	5 (2.5)	0.34
Primary	68 (37.8)	63 (31.2)	
Secondary	82 (45.6)	111 (55)	
Tertiary	25 (13.9)	23 (11.4)	
Nationality, *n* (%)			
Zambian	143 (79.4)	176 (87.1)	0.06
Other	37 (20.6)	26 (12.9)	
Any comorbidity, *n* (%)‡	38 (21.1)	46 (22.8)	0.79
Comorbidity type, *n* (%)			
HIV	22 (12.2)	18 (8.9)	0.38
Hypertension	9 (5.0)	5 (2.5)	0.30
Cardiac disease	2 (1.1)	4 (2.0)	0.79
Diabetes	1 (0.6)	5 (2.5)	1.00
Renal disease	2 (1.1)	2 (1.0)	0.92
Pulmonary disease	1 (0.6)	1 (0.5)	1.00
Tuberculosis	1 (0.6)	2 (1.0)	1.00
Liver disease	1 (0.6)	1 (0.5)	1.00
Other	6 (3.4)	14 (7.1)	0.18
Prior COVID-19, *n* (%)	5 (2.8)	11 (5.4)	0.13
Mask use			
Always or most of the time	97 (53.9)	106 (52.5)	0.14
Sometimes or rarely	51 (28.3)	72 (35.6)	
Don’t use a mask	32 (17.8)	24 (11.9)	
Timing of first vaccine dose, *n* (%)			
≥ 14 days before testing	118 (65.0)	168 (83.2)	< 0.01
0–13 days before testing§	4 (2.2)	4 (2.0)	
After testing	0 (0.0)	0 (0.0)	
Not vaccinated	58 (32.2)	30 (14.9)	
Vaccine type (*N* = 294), *n* (%)			
Janssen (J&J)	110 (90.2)	151 (87.8)	0.75
AstraZeneca	11 (9.0)	20 (11.6)	
Not known	1 (0.8)	1 (0.6)	
Vaccination status, n (%)‖			
Fully	117 (65.0)	166 (82.2)	< 0.01
Partially	0 (0.0)	1 (0.5)	
Indeterminate	5 (2.8)	5 (2.5)	
Unvaccinated	58 (32.2)	30 (14.9)	
Time since vaccination, median days (IQR)	56 (33–91)	53 (27–84)	0.02
Symptomatic, *n* (%)	82 (45.6)	22 (10.9)	< 0.01
Symptoms, *n* (%)			
Cough	68 (37.8)	18 (8.9)	1.00
Rhinorrhea	37 (20.6)	6 (3.0)	0.21
Headache	31 (20.6)	6 (3.0)	0.51
Myalgia	23 (12.8)	2 (1.0)	0.12
Fever	18 (10.0)	3 (1.5)	0.57
Fatigue	10 (5.6)	1 (0.5)	0.52
Sore throat	9 (5.0)	6 (3.0)	1.00
Chills	6 (3.3)	2 (1.0)	1.00
Sinus congestion	3 (1.7)	1 (0.5)	NC
Loss of taste	2 (1.1)	0 (0.0)	NC
Loss of smell	2 (1.1)	0 (0.0)	NC
Others	5 (2.8)	2 (1.0)	NC
Sought health care, n (%)	89 (49.4)	17 (8.4)	< 0.01
Admitted to hospital, n (%)	0 (0.0)	0 (0.0)	NC
Died, *n* (%)	0 (0.0)	0 (0.0)	NC

IQR = interquartile range; NC = not calculated.

* Cases were participants with a positive SARS-CoV-2 rapid diagnostic test (RDT) results and controls were participants with a negative RDT result.

† Student’s *t* test for continuous variables and χ^2^ test or Fisher’s exact test (where any cell size < 10) for categorical variables.

‡ Comorbidities included hypertension, cardiac disease, obesity, pulmonary disease, kidney disease, liver disease, diabetes, cancer, HIV, and tuberculosis (current or past).

§ Participants classified as indeterminate because immune status at time of testing was unknown.

‖ Full vaccination was defined having received the first dose of a one-dose vaccine or second dose of a two-dose vaccine ≥ 14 days before COVID-19 testing. Partial vaccination was defined has having received the first dose of a two-dose vaccine ≥ 14 days before COVID-19 testing but not the second dose or the second dose ≤ 13 days before testing. Persons who received their first dose of a COVID-19 vaccine 0–13 days before testing or had an unknown type of COVID-19 vaccine were considered to have indeterminate vaccination status.

Overall, 294 (77.0%) participants received ≥ 1 COVID-19 vaccine dose; of these 283 (96.3%) were fully vaccinated, 1 (0.3%) partially vaccinated, and 10 (3.4%) indeterminate. Among fully vaccinated participants, 253 (89.4%) received the one-dose Janssen vaccine and 30 (10.6%) received the two-dose AstraZeneca vaccines. None had received an additional (“booster”) vaccine dose. The median time since receipt of a full primary vaccine series was 54 days (interquartile range: 28**–**85 days).

There were 180 (47.1%) COVID-19–positive incarcerated persons (i.e., cases) and 202 (52.9%) COVID-19–negative persons (i.e., controls). Among positive cases, 117 (65.0%) were in fully vaccinated persons (i.e., breakthrough infections), and 166 (82.2%) controls were fully vaccinated. Of the 16 (4.1%) persons reporting prior confirmed COVID-19, five (29.4%) were positive during the outbreak (i.e., reinfections).

Overall, 104 (27.2%) persons reported any COVID-19 symptoms, with a greater proportion among cases (45.6% versus 10.9%; *P* < 0.01, χ^2^ test). The most common symptoms among COVID-19 cases were cough (37.8%), rhinorrhea (20.6%), headache (17.2%), and myalgias (12.8%) (Table 1). Eighty-nine (49.4%) of the 180 participants with cases sought medical care at the prison clinic, and none (0%) were admitted or died. Cases were more likely to have sought health care (49.4% versus 8.4%, *P* < 0.01, χ^2^ test).

Cases were less likely to be fully vaccinated than controls (65.0% versus 82.2%; *P* < 0.01, χ^2^ test) (Table 2). VE against SARS-CoV-2 infection was 64.8% (95% confidence interval [CI]: 36.1–81.0%) and VE against symptomatic SARS-CoV2 infection was 72.9% (95% CI: 42.0–87.5%). VE was higher for those vaccinated within the past 60 days compared with > 60 days before COVID-19 testing, although the confidence intervals overlapped (VE for SARS-CoV-2 infection: 74.6% [95% CI: 50.3–87.4%] versus 54.2% [95% CI: 6.9–77.9%], respectively). The sensitivity analysis excluding symptomatic controls did not meaningfully change the VE estimates (VE against SARS-CoV-2 infection was 64.1% [95% CI: 35.8–80.2%] and VE against symptomatic SARS-CoV2 infection was 73.4% [95% CI: 44.6–87.4%]). Forty persons (10.5%) in the analysis reported being HIV-positive, among whom 77.3% of cases were vaccinated compared with 94.4% of controls (*P* = 0.20, χ^2^ test). Among persons living with HIV (PLHIV), VE against SARS-CoV-2 infection was 82.2% (95% CI: –107.0 to 99.4%).

**Table 2:** COVID-19 vaccine effectiveness against Omicron variant—Zambia, December 2021

Vaccination status	Fully vaccinated, *n* (%)		Vaccine effectiveness, % (95% CI)*	
	Cases	Controls	SARS-CoV-2 infection	Symptomatic infection
Vaccine brand^†^				
Any vaccine (*N* = 371)	117 (66.9)	166 (84.7)	64.8 (36.1–81.0)	72.9 (42.0–87.5)
Janssen (*N* = 341)	106 (64.6)	147 (83.1)	63.6 (33.6–80.5)	73.0 (41.6–87.7)
AstraZeneca (*N* = 118)	11 (15.9)	19 (38.8)	89.4 (59.5–97.8)	85.1 (19.5–98.0)
Vaccination timing†				
≤ 60 days (*N* = 249)	59 (50.4)	102 (77.3)	74.6 (50.3–87.4)	83.7 (60.8–93.6)
> 60 days (*N* = 210)	58 (50.0)	64 (68.1)	54.2 (6.9–77.9)	53.2 (–16.6 to 81.6)

CI: confidence interval

* Adjusted for age, number of comorbidities, jail cell, and mask use.

† Analyses presented for fully vaccinated and unvaccinated persons only. Persons who were partially vaccinated (*N* = 1) or indeterminate status (i.e., received their first dose of a COVID-19 vaccine 0 to 13 days before testing or had an unknown type of COVID-19 vaccine) (*N* = 10) were excluded from analyses.

## DISCUSSION

During a prison COVID-19 outbreak in Zambia, COVID-19 vaccination protected against SARS-CoV-2 infection and symptomatic illness while Omicron was the dominant strain in the country.^1^ Correctional services in Zambia achieved high COVID-19 vaccine coverage among incarcerated persons before the Omicron variant wave through offering of vaccination upon admission to the prison and on-demand vaccination thereafter, and enhanced COVID-19 screening and testing with encouragement of vaccination for those testing negative. These findings provide important local evidence that might help increase COVID-19 vaccination in Zambia, where many more Zambians need to be vaccinated to reach the African Union targets.^9^

The SARS-CoV-2 Omicron variant contains numerous mutations to the spike protein, which may lead to immune evasion. VE estimates reported here are lower compared with prior strains,^10^^,^^11^ and the high proportion of breakthrough infections in this outbreak supports in theory the possibility of immune-evading capability of Omicron. Symptomology was mild among participants, which is consistent with reports from other countries that experienced Omicron surges.^8^^,^^12^ VE for severe illness could not be accessed because no persons were hospitalized or died during this outbreak although other studies have demonstrated durability of this outcome for Omicron.^13^ Similarly, VE of a booster dose could not be assessed because Zambia did not begin offering a booster dose until January 2022. The relatively short time since vaccination might explain the relatively higher effectiveness findings against Omicron.^5^^,^^14^^,^^15^ However, there was a suggestion of waning immunity when comparing the VE point estimates of participants fully vaccinated over 2 months before the outbreak with those vaccinated within the past 2 months.

Although data are still emerging about COVID-19 VE in PLHIV, vaccines are expected to work well based on immunological studies.^16^ Clinical data from South Africa and Russia indicate comparable VE among PLHIV, especially for severe disease.^7^^,^^17^ Zambia has achieved high antiretroviral treatment coverage and HIV viral load suppression, meaning many PLHIV are not immunocompromised. Although VE point estimate among PLHIV was consistent with the other estimates in this study, it was not statistically significant likely because of the small sample size of PLHIV.

This study had several limitations. All cases were confirmed with RDTs, which have lower sensitivity than PCR tests.^18^ Additionally, beyond initial test results, serial testing was not available, meaning that some controls might have been in their incubation period at the time of testing and therefore were misclassified. Although Omicron was already dominant in Zambia before this outbreak occurred,^19^ it was not confirmed by genomic sequencing in this outbreak. Lastly, although few participants reported a prior confirmed SARS-CoV-2 infection, the actual number might be much higher considering only a small proportion of cases are confirmed in Zambia.^20^

Rapid investigation of an outbreak in a closed setting demonstrated VE of COVID-19 vaccines against Omicron infection in Zambia. COVID-19 vaccination remains a critical tool in decreasing SARS-CoV-2 transmission and severity especially when coupled with a layered prevention including well-fitting facemask use, hand hygiene, limiting large gatherings, and adequate ventilation and/or outdoor gatherings. Continuing to scale up COVID-19 vaccination rapidly to all eligible persons in Zambia can help prevent SARS-CoV-2 transmission and symptomatic COVID-19.
